# The UQTR university-based chiropractic clinic cohort (UQTRChiCo): description and summary of an available data source for research collaborations

**DOI:** 10.1186/s12998-026-00642-2

**Published:** 2026-05-20

**Authors:** Marc-André Blanchette, Cesar A. Hincapié, Jean Théroux, Lisanne Guérin, Sandra Basta, Arianne Bezeau, Jean-Luc Gauthier, André Bussières

**Affiliations:** 1 Département chiropratique, Université du Québec à Trois-Rivières, 3351 boul. des Forges, C.P. 500, Trois- Rivières, QC G9A 5H7 Canada; 2 Département des sciences de la santé, Université du Québec en Abitibi- Témiscamingue (UQAT), Rouyn-Noranda, QC Canada; 3 Musculoskeletal Epidemiology Research Group–Epidemiology, Biostatistics and Prevention Institute (EBPI), University of Zurich, Zurich, Switzerland; 4 Dalla Lana School of Public Health, University of Toronto, Toronto, Canada; 5 CHU Sainte-Justine Research Center, Montreal, QC Canada; 6 Faculty of Medicine and Health Sciences, McGill University, Montreal, QC Canada

**Keywords:** Chiropractic education, Clinical practice guidelines, Cata resource, Musculoskeletal disorders, Quality of care, Academic clinic cohort

## Abstract

**Background:**

Musculoskeletal disorders are the leading cause of disability in Canada, and chiropractic care is among the most commonly used healthcare services for their management. Despite this, little is known about the quality of care and adherence to clinical practice guidelines in chiropractic educational settings. This study aimed to describe the Université du Québec à Trois-Rivières university-based chiropractic clinic cohort (UQTRChiCo) and summarize its patient characteristics, care provided, and available outcome data.

**Methods:**

We conducted a retrospective cohort study of patients who first consulted at the Université du Québec à Trois-Rivières (UQTR) chiropractic clinic in 2017, with longitudinal follow-up through 2019. Reasons for clinical consultation (i.e., specific chief complaints) were treated as separate observations. Data were extracted from clinical records and included patient demographics, clinical presentations, care provided, and outcomes measured using validated questionnaires. Follow-up assessments were obtained from re-evaluations conducted at multiple time points between 1 January 2017 and 31 December 2019.

**Results:**

The UQTRChiCo comprises 2,148 distinct reasons for consultation from 1,305 unique patients (59% female, mean age 31 ± 18 years). The most common presentations were lumbar spine (28%), cervical spine (18%), and thoracic spine complaints (10%). Multiple concurrent complaints were reported in 44% of patients, and 43% of conditions were chronic. Care commonly included spinal manipulative therapy (88%) and soft tissue treatments (93%). Patient education and exercise prescription were documented in 67% and 48% of consultations, respectively. Diagnostic imaging was performed in 19% of consultations. Follow-up outcome documentation was available for 58% of consultations at the first re-evaluation, decreasing to 16% by the third re-evaluation.

**Conclusion:**

The UQTRChiCo provides a comprehensive description of an academic chiropractic clinical population primarily seeking care for spinal musculoskeletal complaints. Care delivery reflected a multimodal approach with restrained use of diagnostic imaging. While short-term outcome monitoring proved feasible, substantial attrition over time underscores a key challenge for longitudinal practice-based research in educational clinics. The UQTRChiCo represents a valuable resource for advancing research on care quality, guideline adherence, and patient outcomes in chiropractic training environments.

**Supplementary Information:**

The online version contains supplementary material available at 10.1186/s12998-026-00642-2.

## Background

Musculoskeletal disorders are the leading cause of years lived with disability globally and impose a significant burden on healthcare systems [1]. In Canada, these conditions are the primary source of disability, and chiropractic care is among the most commonly utilized healthcare services for their management [2]. Despite its widespread utilization, important knowledge gaps remain regarding the quality of care delivered in chiropractic educational training environments where future practitioners develop clinical reasoning and practice habits.

High-quality clinical practice guidelines on the management of musculoskeletal disorders consistently recommend patient education, self-care advice, exercise, manual therapy, and, when necessary, short courses of nonsteroidal anti-inflammatory drugs (NSAIDs) and other conservative approaches [3]. Although most patients with low back pain improve substantially within the first month, symptoms often recur or become persistent [4]. Recent evidence suggests that the quality and timing of initial care greatly affects recovery, and overly aggressive early management can contribute to iatrogenic harm [5–7]. Consequently, more judicious use of diagnostic imaging and pharmacological interventions such as opioids is advised [8].

Despite available high-quality clinical practice guidelines, considerable variation persists in how these high burden conditions are managed across healthcare disciplines, including chiropractic [9–19]. Multiple factors, including clinician’s knowledge, attitudes, beliefs, and skills, patient preferences and characteristics, organizational and educational contexts, and broader system-level influences shape implementation of evidence-based care [20–22].

Introducing and reinforcing evidence-based practice (EBP) early in professional training may be more effective than attempting to modify entrenched behaviours in practice [23]. Yet, little is known about the type and quality of care delivered in chiropractic teaching clinics globally [24–26]. Published studies from chiropractic teaching settings are limited in scope, often focusing on describing patient characteristics rather than evaluating processes, guideline adherence, or care outcomes. Consequently, there is a lack of robust data to assess how well training environments model evidence-informed practice and whether the care delivered aligns with contemporary musculoskeletal practice guidelines. A systematic understanding of these care patterns is crucial for several reasons. First, it allows educators and regulators to identify areas where guideline adherence can be strengthened, ensuring that emerging clinicians are equipped to provide high-value, evidence-based care. Second, linking care processes to outcomes within a teaching context helps clarify how educational supervision and clinic structure influence patient recovery and service quality. Finally, the availability of structured, longitudinal data can facilitate research collaborations aimed at improving musculoskeletal care delivery more broadly.

The UQTR University-based Chiropractic Clinic Cohort (UQTRChiCo) was established to address these gaps by systematically capturing real-world patient care from a supervised academic clinic. The purpose of this study was to describe the UQTR University-based Chiropractic Clinic Cohort (UQTRChiCo) and to summarize the characteristics of this population, including patient demographics, care provided, and available outcome data, informing the scientific community of the availability of these data as a resource for research collaborations.

By documenting the real-world patterns of care and outcomes in a university clinic, the UQTRChiCo provides a foundation for understanding how the care delivered by chiropractic interns translates into patient outcomes.

## Methods

### Cohort description

#### Study design and setting

The UQTRChiCo is a retrospective observational study. Patients who had their first consultation at the UQTR University Chiropractic Clinic between 1 January 2017 and 31 December 2017, were selected. The UQTRChiCo database is structured by reason for consultation, defined as a unique clinical presentation or chief complaint recorded during an initial or follow-up consultation. All reasons for consultation documented between 1 January 2017 and 31 December 2019, were extracted. Each reason for consultation represents a distinct observation, even when multiple reasons for consultation are documented for the same patient during the same or different visits (e.g., concurrent neck and low back pain). While distinct reasons for consultation within a patient may be correlated [27] (e.g., neck pain intensity may influence low back pain intensity), this reason for consultation-based structure allows for condition-specific analysis while reflecting the complexity of patient presentations commonly encountered in clinical practice. According to the UQTR university chiropractic clinic policy, patients can have a maximum of three concurrently active reasons for consultation; previous clinical conditions must be closed before new ones can be opened.

Data collection extended from the initial consultation through 31 December 2019, providing a follow-up period of 2 to 3 years per patient depending on their consultation date. This timeframe was selected to provide a stable period for analyzing care patterns and outcomes, as it precedes the COVID-19 pandemic and the subsequent transition to a new electronic record system. The UQTR chiropractic clinic operates as a supervised teaching facility where chiropractic interns deliver patient care under the direct supervision of licensed chiropractors. All care provided adheres to university clinical protocols and provincial regulatory standards.

This study received ethics approval from the UQTR Research Ethics Board (CER-20-266-07.05). All procedures were conducted in accordance with institutional guidelines and applicable privacy legislation.

#### Inclusion and exclusion criteria

All reasons for consultation from patients who initiated care at the UQTR chiropractic clinic in 2017 were eligible for inclusion if their clinical records were complete and available for data extraction until the end of 2019. There were no explicit exclusion criteria except for incomplete records that were not available for data extraction.

## Data collection

### Data sources and collection

At the university clinic, clinical information is gathered from multiple sources: patients, treating interns (4th and 5th year), supervising clinicians, and interprofessional communications with external healthcare providers (e.g., referring physicians, co-management practitioners). At intake, patients are asked to provide sociodemographic details (age, sex,…), detailed medical histories, and complete a validated Patient-Reported Outcome Measures (PROMs) including the Visual Analog Scale [VAS] and the relevant condition-specific measures: Oswestry Disability Index (low back pain), [28] Neck Disability Index NDI), [29] Disabilities of Arm Shoulder and Hand questionnaire (DASH), [29,30] and Lower Extremity Functional Scale (LEFS) [31]. All PROMs scores are converted to a 0 to 100% score to facilitate comparison.

### Clinical documentation

After completing required forms for initial interviews and physical examinations, chiropractic interns prepare case summaries that include detailed treatment plans, differential diagnoses, treatment modalities, patient recommendations, and prognoses. Supervising clinicians then review the findings of the clinical interviews and examination in patient records. Alongside interns, they document related comorbidities and presence of indicators of serious pathologies or psychological risks of delayed recovery (i.e., red and yellow flags) to determine existing contraindications to chiropractic care. Supervising clinicians must approve all information entered by interns in patient records. Copies of all written communications with other healthcare professionals are kept in patient files.

For each visit, interns complete SOAP (Subjective, Objective, Assessment, Plan) progress notes [32]. Students are encouraged to regularly record pain levels and/or average improvement ratings on a 0-100% verbal rating scale in the subjective section. At each re-evaluation, interns’ complete forms detailing new physical examination findings, treatment plans, and prognoses.

### Data extraction and quality assurance

A standardized data extraction form was developed iteratively prior to full-scale extraction. A preliminary version of the extraction grid was piloted by the three extractors (LG, SB, ABé) and an experienced clinician (J-LG) on 120 patient records. Following this pilot phase, a group consensus meeting was held to review coding decisions, resolve ambiguities, and refine variable definitions, resulting in an enhanced extraction grid and a finalized codebook used for all subsequent extraction.

Throughout the extraction period, periodic team meetings, ranging from monthly to weekly depending on extraction workload, were held to compare descriptive statistics across records extracted by the four extractors. These meetings served to identify unexpected clinical contexts that introduced variability in coding decisions, and to establish consensus on how such cases should be handled going forward. Where new coding rules were established, previously extracted records affected by these decisions were revisited and corrected accordingly. M-AB provided ongoing methodological oversight throughout this process, reviewing extraction outputs for accuracy and consistency across all variables. We include a description of all variables and their data sources in the Supplementary file 1.

### Variables and outcome measures

*Patient demographics*: Age, sex, occupation (National Occupation Code), [33] socioeconomic factors, reasons for consultation (11th International Classification of Disease), [34] neurological signs, previous care received, comorbidities, context of injury, and contraindications to care.

*Care provided*: Dates of all chiropractic consultations, condition opening and closure dates, treatment modalities provided, number and frequency of visits, and referrals to other providers.

*Outcomes data*: Scores from validated PROMs and their assessment dates, quantitative evaluations of pain (VAS) at rest and during activity, as well as the perceived percentage of global improvement recorded in the SOAP notes.

*Quality of care*: For common musculoskeletal conditions, recent recommendations from the Canadian Chiropractic Guideline Initiative were operationalized into criteria assessable from patient records (Supplementary file 1) [35,36]. The extractor indicated whether each criterion was met based on clinical judgment after consulting the complete patient file. The validation of these criteria will be covered in future publications.

### Analysis of data

All analyses were conducted at the reason for consultation-level using IBM SPSS version 29.0.2.0 (Armonk, NY: IBM Corp). For patient-specific variables (e.g., age and sex), estimates represent the distribution of unique patients. For reason for consultation-specific variables (e.g., pain intensity, functional scores, treatment approaches), analyses were conducted at the reason for consultation level to capture condition-specific clinical patterns while acknowledging that some patients contributed multiple reason for consultation.

#### Patient demographics and clinical profile 

Continuous variables are presented as means with standard deviations (SD), and categorical variables as frequencies and proportions. Demographic and clinical characteristics are described for the full sample. Data completeness is reported for all core variables.

#### Care provided

Treatment modalities, diagnostic imaging utilization, number and frequency of visits, patient education, and referral patterns are described at the reason for consultation-level using frequencies and proportions. Imaging utilization rates and documented clinical justifications are presented to characterize care delivery patterns within the academic clinic.

#### Available outcome data

Bivariate comparisons of pain intensity and functional outcome scores between initial assessment and first re-evaluation were performed using paired t-tests with statistical significance set at *p* < 0.05. Non-parametric alternatives (Wilcoxon signed-rank test) were run as a sensitivity analysis considering sample size limitation. We report the proportion of reasons for consultation with documented outcome data at each re-evaluation timepoint (first, second, and third) to describe the availability and completeness of longitudinal outcome data within the UQTRChiCo dataset.

## Results

**Fig. 1:**
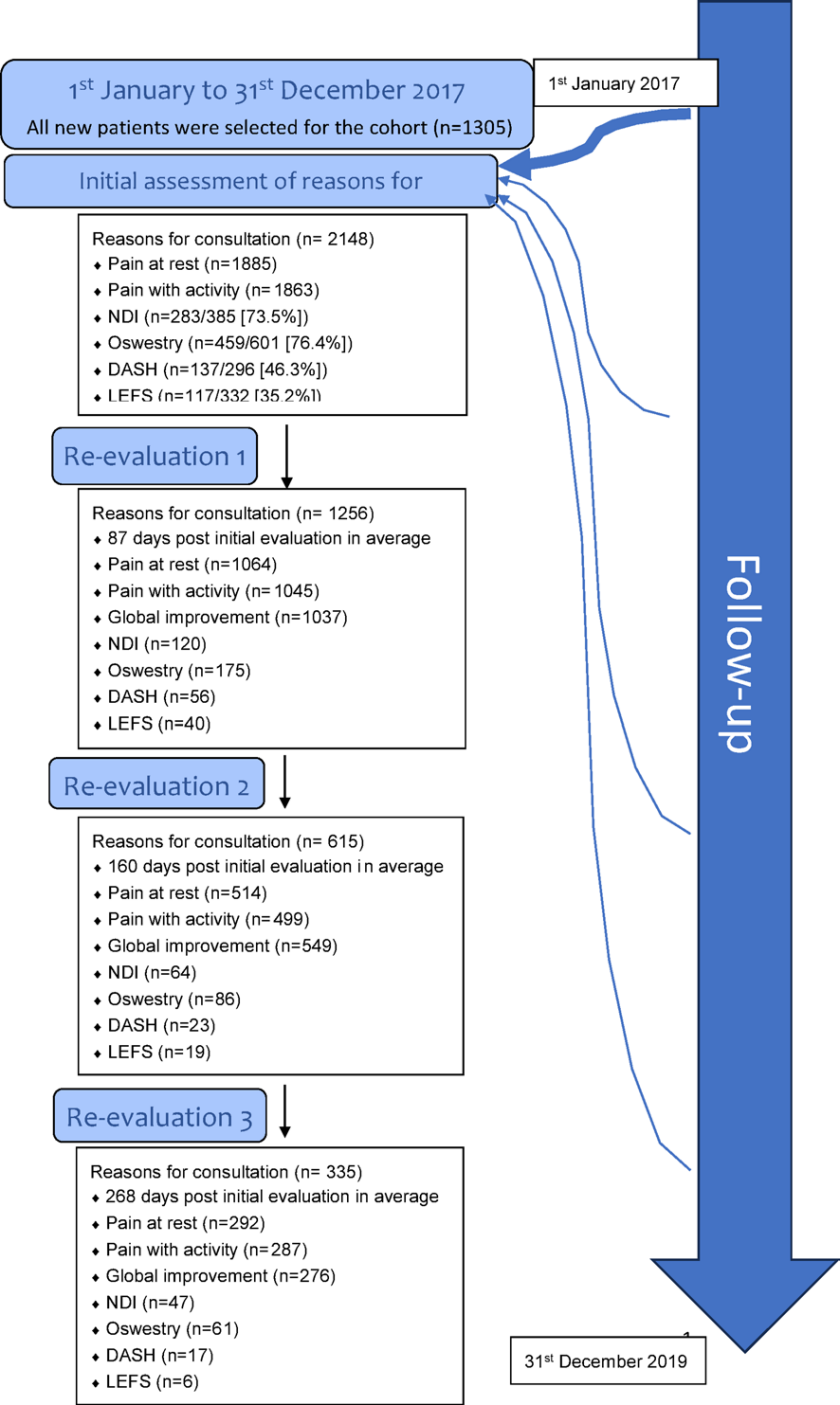
Flow diagram of UQTRChiCo

The UQTRChiCo comprises 1,305 unique patients contributing 2,148 distinct reasons for consultation followed until 31 December 2019. Figure 1 details the flow of unique patients and reasons for consultation in the UQTRChiCo. Core demographic and clinical variables were comprehensively documented with minimal missing data (Table 1).

**Table 1:** Demographic and clinical characteristics of UQTRChiCo reasons for consultations during their initial assessment (*N* = 2,148)

		*n* (%) Unless indicated otherwise
Patient demographics*	Age, years (Mean (SD))	31.0 (17.5)
	Age categories	
	0–17 years	128 (9.8)
	18–25 years	550 (42.2)
	26–40 years	332 (25.5)
	41–65 years	214 (16.4)
	> 65 years	80 (6.1)
	Sex	
	Female	767 (58.8)
	Male	538 (41.2)
Cohort structure	Total reasons for consultation	2,148 (100.0)
	Unique patients*	1,305
	Patients with multiple reasons for consultations*	44.4%
Chief complaint location	Lumbar spine	601 (28.0)
	Cervical spine	385 (17.9)
	Thoracic spine	223 (10.4)
	Upper extremity	296 (13.8)
	Lower extremity	332 (15.5)
	Screening/Preventive	154 (7.2)
	Other locations	157 (7.3)
Reason for consultation duration	Acute (1–7 days)	702 (32.7)
	Sub-acute (1 week − 3 months)	126 (5.8)
	Recurrent	190 (8.8)
	Chronic (> 3 months)	923 (42.9)
Pain and disability scores Mean (SD)	VAS pain at rest (0–10)	2.1 (2.5)
	VAS pain with activity (0–10)	5.8 (2.5)
	NDI score (%) (*n* = 283)	22.4 (12.6)
	Oswestry score (%) (*n* = 459)	30.5 (16.7)
	DASH score (%) (*n* = 137)	32.5 (20.6)
	LEFS score (%) (*n* = 117)	46.8 (31.3)
Initial clinical history	Current medication use documented	1,141 (53.1)
	Pain medication	230 (10.7)
	Hypertension medication	137 (6.4)
	Depression medication	139 (6.5)
	Previous healthcare consultation	852 (39.7)
	Previous chiropractic care	348 (16.2)
	Previous physiotherapy	190 (8.8)
Red flags present	Nocturnal pain	186 (8.7)
	Unexplained weight loss	27 (1.3)
	Fever	14 (0.7)
	Morning stiffness†	156 (7.3)
	Bladder/bowel changes	16 (0.7)
	Saddle anesthesia	4 (0.2)

UQTRChiCo, UQTR University-based chiropractic clinic cohort; VAS, Visual analog scale; NDI, Neck disability index; DASH, Disabilities of arm, shoulder and hand; LEFS, Lower extremity functional scale

† Morning stiffness (> 30 min duration) is included as a potential indicator of rheumatologic conditions in the UQTR clinic screening protocol

* Analysis reported for the 1,305 patients

### Patient demographics and clinical profile

The cohort was predominantly young (mean age 31 ± 18 years), with a slight female majority (59%), and spanned a broad age range from paediatric to older adult patients. Spinal complaints were the most common reason for consultation, with lumbar (28%), cervical (18%), and thoracic (10%) regions predominating. Chronic presentations accounted for 43% of reasons for consultation, and 44% of patients managed multiple concurrent reasons for consultation during the study period. At intake, VAS pain scores averaged 2.1 ± 2.5 at rest and 5.8 ± 2.5 during activity. Condition-specific functional scores are summarized in Table 1.

### Care provided

The mean number of treatments per clinical condition was 9.6 ± 10.4. Spinal manipulative therapy and soft tissue treatments were delivered in 88% and 93% of consultations, respectively. Patient education and exercise prescription were documented in 67% and 48% of consultations. Diagnostic imaging was performed in 19% of consultations, with clinical justifications documented in most cases. Referral to other healthcare professionals was infrequent. Full details of treatment variables and data completeness are provided in Table 2.

**Table 2:** Available data sources and variables in UQTRChiCo (*N* = 2,148)

Data category	Variables available	Data completeness (%)
*Clinical assessment*		
Chief complaint location	Anatomical region classification	100.0
Episode duration	Days since onset	80.6
Episode classification	Acute/Sub-acute/Recurrent/Chronic	90.5
Pain radiation	Yes/No	92.9
VAS pain at rest	0–10 scale (intake/R1/R2/R3)	87.8 / 49.5 / 23.9 / 13.6
VAS pain with activity	0–10 scale (intake/R1/R2/R3)	86.7 / 48.6 / 23.2 / 13.4
Functional assessments**		
Neck Disability Index	0-100% disability (intake/R1/R2/R3)	73.5 / 31.2 / 16.6 / 12.2
Oswestry Disability Index	0-100% disability(intake/R1/R2/R3)	76.4 / 29.1 / 14.3 / 10.1
DASH questionnaire	0-100% disability (intake/R1/R2/R3)	46.3 / 18.9 / 7.8 / 5.7
LEFS questionnaire	0-100% function (intake/R1/R2/R3)	35.2 / 12.0 / 5.7 / 1.8
*Initial physical examination*		
Range of motion assessment	Global/segmental evaluation	95.8
Neurological examination	Reflexes, sensation, motor	90.6
Orthopedic tests	Kemps, Spurling, SLR, etc.	67.0
*Initial medical history*		
Current medications	By category (14 categories)	99.3
Previous healthcare	Type and provider	98.9
Comorbidities	Yes/No with specification	99.2
Red flags	6 categories assessed	97.0
Contraindications	Absolute/relative/region	99.3
*Treatment variables*		
Treatment modalities	15 different categories	98.9
Patient education	11 different types	98.8
Therapeutic estimates	Initial benefit prediction	95.0
Number of treatments	Total count per episode	99.3
Treatment phase	Relief/Correction/Support/Wellness	98.2
imaging data*		
Imaging performed	Yes/No with justification	99.3
Imaging results	15 result categories	98.3*
Clinical indication	9 indications categories	80.3*
*Follow-up assessments*		
Re-evaluation 1	*n* = 1256	58.5
Re-evaluation 2	*n* = 615	28.6
Re-evaluation 3	*n* = 335	15.6
*Complexity indicators*		
Case complexity	Yes/No assessment	99.3
Yellow flags	Psychosocial factors	99.2
Blue/black flags	Occupational factors	99.2

*Only applicable to patients who received imaging

**Only applicable to patients who had the relevant clinical conditions

Data completeness percentages represent the proportion of reasons for consultation for whom the specific variable was documented in the clinical records

### Available outcome data

First follow-up assessments were conducted for 1,246 reasons for consultation (58%), second follow-up for 615 (29%), and third follow-up for 335 (16%). Consequently, pain outcome availability declined progressively across re-evaluation timepoints: VAS measures were documented for approximately 90% of reasons for consultation at intake, 50% at first re-evaluation, 25% at second, and 15% at third (Table 2). Relevant PROMs were documented in about two third of relevant reason for consultations at intake, one quarter at the first re-evaluation and declining to about 10% at the subsequent re-evaluations. The timing of re-evaluations, occurring on average at 87, 160, and 268 days after initial assessment, reflects routine clinical follow-up patterns rather than a standardized protocol, which should be considered when interpreting longitudinal outcome data. Among consultations with available follow-up data, clinically meaningful improvements in pain and functional outcomes were observed between initial assessment and first re-evaluation across all validated outcome measures (Table 3). The non-parametric analysis yielded identical conclusion. Global perceived improvement averaged 69.9% at first re-evaluation, consistent with expected recovery trajectories for musculoskeletal conditions managed in primary care settings.

**Table 3:** Clinical outcomes

Outcome	Initial assessment (Mean ± SD)	First re-evaluation (Mean ± SD)	*n*	Mean difference (95% CI)
VAS at rest	2.0 ± 2.5	1.2 ± 2.0	1007	0.8 (0.7-1.0)
VAS during activity	5.7 ± 2.4	3.5 ± 3.3	978	2.2 (1.9–2.4)
NDI	22.5 ± 11.9	13.3 ± 11.2	155	9.3 (7.5–11.0)
Oswestry	29.7 ± 15.3	19.3 ± 15.2	179	10.5 (8.4–12.5)
DASH	34.3 ± 18.5	24.5 ± 18.8	44	9.8 (4.6–15.0)
LEFS	48.5 ± 31.2	44.6 ± 39.2	26	3.9 (− 6.3-14.2)
Subjective % of improvement of their condition	–	69.9 ± 30.0	1037	–

VAS, Visual analog scale; NDI, Neck disability index; DASH, Disabilities of arm, shoulder and hand; LEFS, Lower Extremity Functional Scale; Oswestry, Oswestry disability index; SD, Standard deviation; CI, Confidence Interval

## Discussion

### Summary of main findings

The UQTRChiCo provides a comprehensive description of patient characteristics, care delivery, and available outcome data from a supervised academic chiropractic teaching clinic. This cohort of 2,148 reasons for consultation from 1,305 unique patients demonstrates that systematic clinical data collection is achievable in an academic teaching environment, with core demographic and clinical variables documented with minimal missing data, treatment variables comprehensively captured across the large majority of consultations, and outcome data available for half of the reasons for consultations at first re-evaluation. Taken together, these characteristics establish the UQTRChiCo as a viable and sufficiently complete data source for future research on chiropractic care in university-based educational clinic settings. The UQTRChiCo patient population is broadly consistent with profiles reported in other academic chiropractic teaching clinic studies in terms of sex distribution, most common complaint types, and case mix complexity [37–39]. The most notable demographic distinction was age. The UQTRChiCo patients were substantially younger than those reported in comparable academic settings, likely reflecting the student population served and the lower-cost care model [37, 38]. Spinal complaints predominated across similar studies [37, 38], and the high prevalence of multiple concurrent reasons for consultation likely reflects the multisite nature of musculoskeletal complaints.

One pattern that differed from other university-based chiropractic clinic studies was the proportion of chronic presentations. Chronic conditions were less common in the UQTRChiCo compared to chiropractic educational clinic cohorts in Switzerland, [38] Canada, [39] and Mexico [37] (43% vs. 57% vs. 67% vs. 76%, respectively). The factors underlying this gradient are unclear and likely multifactorial. The younger age profile of the UQTRChiCo population may partly explain this difference. Differences in care access models, referral pathways, and local patient populations may all contribute. For instance, the Swiss clinic operated within a mandatory universal insurance framework and included a substantial proportion of internally referred patients from spine surgery, which may result in more complex and chronic presentations.

Care delivery in the UQTRChiCo was predominantly multimodal, consistent with current musculoskeletal practice guidelines recommending manual therapy as an adjunct to active approaches rather than as a standalone intervention [3]. Imaging utilization was restrained, suggesting that the supervisory structure of the academic clinic supports appropriate diagnostic decision-making [36, 40]. Nevertheless, the modest proportion of cases documenting patient education and advice on exercise, approximately two-thirds, is an observation that warrants further investigation in future guideline adherence studies using this dataset to understand possible reasons for lower guideline adherence.

Longitudinal outcome data were available for most reasons for consultation at first re-evaluation, with progressive attrition at subsequent timepoints reflecting the practical realities of data collection in real-world clinical workflows. The timing of re-evaluations reflects routine clinical follow-up patterns rather than a standardized research protocol, which should be considered when interpreting longitudinal outcome data. These data can support future investigations of outcome trajectories, predictors of recovery, and the relationship between care processes and patient outcomes within this cohort. The breadth and completeness of the UQTRChiCo data collection, spanning patient demographics, care processes, clinical justifications, and outcome measures, establishes this cohort as a viable resource for future research on guideline adherence and determinants of clinical decision-making in academic chiropractic education. The variability observed across key clinical decisions, including imaging utilization, patient education, exercise prescription and treatment selection, confirms that sufficient heterogeneity exists within the dataset to support meaningful analytical studies.

### Strength and limitations

The reason-for-consultation structure of the UQTRChiCo closely mirrors clinical reality. It allows condition-specific analyses while capturing the complexity of patients managing multiple concurrent complaints. This feature is particularly advantageous for educational research examining how interns learn to manage diverse presentations. The dataset also represents a stable historical window (2017–2019) predating both the COVID-19 pandemic and the clinic’s transition to a new electronic records system. It therefore provides a coherent and internally consistent snapshot of academic chiropractic practice that is well-suited for secondary analyses and research collaborations.

Several limitations should be acknowledged. First, the single-center design and the healthcare system factors specific to Québec should be considered when interpreting findings in other clinical, educational, or jurisdictional contexts. Second, the retrospective design raises concerns regarding data quality. Clinical documentation was not intended for research purposes and varies in completeness and consistency across supervising clinicians and interns. Despite standardized extraction protocols and the iterative quality assurance process described above, some variables may have been incompletely recorded or inconsistently interpreted across the extraction period [41]. The clinic’s administrative policy restricts the number of concurrently active reasons for consultation to a maximum of three. Patients presenting with more than three concurrent complaints may therefore be under-represented, which could lead to an underestimation of the true prevalence of multimorbidity in this population. Third, loss to follow-up limits the completeness of longitudinal outcome data. Attrition in this type of clinical cohort is difficult to interpret directionally, as both patients who fully recovered and those who sought care elsewhere may be underrepresented at later timepoints. Findings based on follow-up data should therefore be interpreted with caution. Finally, quality assurance was conducted by the principal investigator, who was also responsible for supervising the data extraction process. Although this was partially mitigated by the iterative group consensus process involving all extractors, independent external validation was not performed.

### Future directions

The current UQTRChiCo dataset provides immediate opportunities for investigating specific clinical questions within the academic chiropractic setting, including the development of prediction models for treatment outcomes, evaluation of care patterns across patient subgroups, and formal assessment of adherence to clinical practice guidelines using the operationalized criteria described in this paper. The variability documented across key clinical decisions provides the analytical conditions necessary for these investigations to yield meaningful findings.

## Conclusion

The UQTRChiCo describes a clinically diverse patient population seeking care at a supervised academic chiropractic teaching clinic, with comprehensive documentation of demographics, care delivery, and clinical outcomes across 2,148 reasons for consultation from 1,305 unique patients. Core variables were captured with minimal missing data at intake, treatment processes were systematically documented across the large majority of consultations, and outcome data were available for half of consultations at first re-evaluation, collectively establishing UQTRChiCo as a sufficiently complete and internally consistent data resource. This 2017–2019 cohort captures a stable period of academic chiropractic practice predating the COVID-19 pandemic and subsequent changes to the clinic’s documentation system and serves as a viable foundation for future research on care quality, guideline adherence, and clinical outcomes in academic chiropractic education.

## Supplementary Information

Below is the link to the electronic supplementary material.

Supplementary Material 1

## Data Availability

The UQTRCo dataset is available as a data resource for collaborative research projects, subject to obtaining appropriate ethical approvals and entering into data sharing agreements. This dataset offers opportunities for investigating specific clinical questions, creating prediction models for treatment outcomes, evaluating various treatment approaches within the academic setting, and conducting comparative analyses with other academic or community-based chiropractic cohorts. Interested researchers are encouraged to contact the corresponding author to explore potential collaborations.

## Declarations

### Use of artificial intelligence tools

During the preparation of this manuscript, the authors used Claude Sonnet 4.6 (Anthropic, 2026) to assist with text editing and language refinement. All content was subsequently reviewed and verified by the authors, who take full responsibility for the accuracy and integrity of the published work.
